# “Two-Cell Assemblage” Assay: A Simple *in vitro* Method for Screening Hair Growth-Promoting Compounds

**DOI:** 10.3389/fcell.2020.581528

**Published:** 2020-11-24

**Authors:** Sunhyae Jang, Jungyoon Ohn, Bo Mi Kang, Minji Park, Kyu Han Kim, Ohsang Kwon

**Affiliations:** ^1^Laboratory of Cutaneous Aging and Hair Research, Clinical Research Institute, Seoul National University Hospital, Seoul, South Korea; ^2^Institute of Human Environment Interface Biology, Seoul National University, Seoul, South Korea; ^3^Department of Dermatology, Seoul National University College of Medicine, Seoul, South Korea

**Keywords:** alopecia, hair follicle, *in vitro* assay, outer root sheath cell, screening assay, dermal papilla cell

## Abstract

Alopecia arises due to inadequate hair follicle (HF) stem cell activation or proliferation, resulting in prolongation of the telogen phase of the hair cycle. Increasing therapeutic and cosmetic demand for alleviating alopecia has driven research toward the discovery or synthesis of novel compounds that can promote hair growth by inducing HF stem cell activation or proliferation and initiating the anagen phase. Although several methods for evaluating the hair growth-promoting effects of candidate compounds are being used, most of these methods are difficult to use for large scale simultaneous screening of various compounds. Herein, we introduce a simple and reliable *in vitro* assay for the simultaneous screening of the hair growth-promoting effects of candidate compounds on a large scale. In this study, we first established a 3D co-culture system of human dermal papilla (hDP) cells and human outer root sheath (hORS) cells in an ultra-low attachment 96-well plate, where the two cell types constituted a polar elongated structure, named “two-cell assemblage (TCA).” We observed that the long axis length of the TCA gradually increased for 5 days, maintaining biological functional integrity as reflected by the increased expression levels of hair growth-associated genes after treatment with hair growth-promoting molecules. Interestingly, the elongation of the TCA was more prominent following treatment with the hair growth-promoting molecules (which occurred in a dose-dependent manner), compared to the control group (*p* < 0.05). Accordingly, we set the long axis length of the TCA as an endpoint of this assay, using a micro confocal high-content imaging system to measure the length, which can provide reproducible and reliable results in an adequate timescale. The advantages of this assay are: (i) it is physiologically and practically advantageous as it uses 3D cultured two-type human cells which are easily available; (ii) it is simple as it uses length as the only endpoint; and (iii) it is a high throughput system, which screens various compounds simultaneously. In conclusion, the “TCA” assay could serve as an easy and reliable method to validate the hair growth-promoting effect of a large volume of library molecules.

## Introduction

Each mammalian hair follicle (HF) is a mini organ that undergoes regenerative cycling, consisting of the following phases: telogen (quiescence), anagen (regeneration), and catagen (degeneration) (Lei and Chuong, 2016). Inadequate HF stem cell activation or proliferation with telogen phase prolongation causes alopecia (Lei and Chuong, 2016). This condition can present in numerous pathologies, such as premature aging, overt hormonal effects, or drug side effects (Price, 1999). In terms of quality of life, alopecia is a distressing event affecting millions of people worldwide (Hunt and Mchale, 2005), potentially causing severe negative psychological effects (Cash, 1992).

To date, two FDA approved drugs, finasteride and minoxidil (MNX), are used to treat patients with certain types of alopecia in respect of modulating the HF regeneration cycle and promoting hair growth (Rittmaster, 1994; Messenger and Rundegren, 2004). However, new molecules that can effectively treat alopecia are needed, considering the limited usage range and/or ineffectiveness of finasteride and MNX in a proportion of patients. The increasing therapeutic and cosmetic demand for the alleviation of alopecia has driven research toward the discovery or synthesis of novel compounds that can promote hair growth by mirroring HF stem cell activation or proliferation and initiating the anagen phase.

To date, several methods for evaluating the hair growth-promoting effects of candidate compounds have been used: *in vitro* human dermal papilla (hDP) and human outer root sheath (hORS) cell assays, *ex vivo* human HF (hHF) organ culture, and animal models (Ohn et al., 2019). However, most of these methods are difficult to use for large scale simultaneous screening of various compounds, because of the limited availability of hHFs (Havlickova et al., 2009). In order to screen the hair growth-promoting efficacy of candidate substances in a large molecular library scale, an easily obtainable and accessible platform is essential. Herein, we introduce a simple and reliable *in vitro* assay, named the “two-cell assemblage (TCA)” assay, using two types of hHF-constituting cells, hDP and hORS, for the simultaneous screening of the hair growth-promoting effects of candidate compounds on a large scale.

## Materials and Methods

### Ethics Statement

This study protocol was approved by the Institutional Review Board of Seoul National University (2005-067-1124), adhering to Helsinki guidelines. All subjects provided written informed consent before scalp skin tissues were taken.

### hDP and hORS Cells Preparation

Healthy young Koreans, with no obvious scalp diseases, provided occipital scalp skin tissue for this study. From the tissue, hHFs were isolated and dissected into single follicular units with a No. 20 blade under a stereomicroscope (Olympus). The micro-dissected hHFs were used for primary hDP and hORS cell cultures. The hDP cells were cultured as described previously (Magerl et al., 2002). In short, candlelight-shaped dermal papilla (DP) of HFs were dissociated in Dulbecco’s Modified Eagle’s Medium (Welgene) supplemented with 10% fetal bovine serum (Welgene) and antibiotic/antimycotic solution (penicillin and streptomycin; Gibco). For hORS cells, the hair shaft and bulb region of the dissected hHF were cut off and immersed in DMEM supplemented with 20% FBS, as described by Choi et al. (2019). On the third day, the medium was changed to Epilife medium (Gibco) supplemented with human keratinocyte growth supplement (Gibco) and antibiotics/antimycotics. We used a fluorochrome (Cell Tracker CM-DiI Dye or Qtracker 525 Cell Labeling Kit, Invitrogen), in accordance with the manufacturer’s instruction, to label the cultured hORS or hDP cells, respectively. All cells were kept in a humidified 5% CO_2_ atmosphere at 37°C.

### TCA Structure Preparation and Treatment With Hair Growth-Promoting Molecules

**FIGURE 1:**
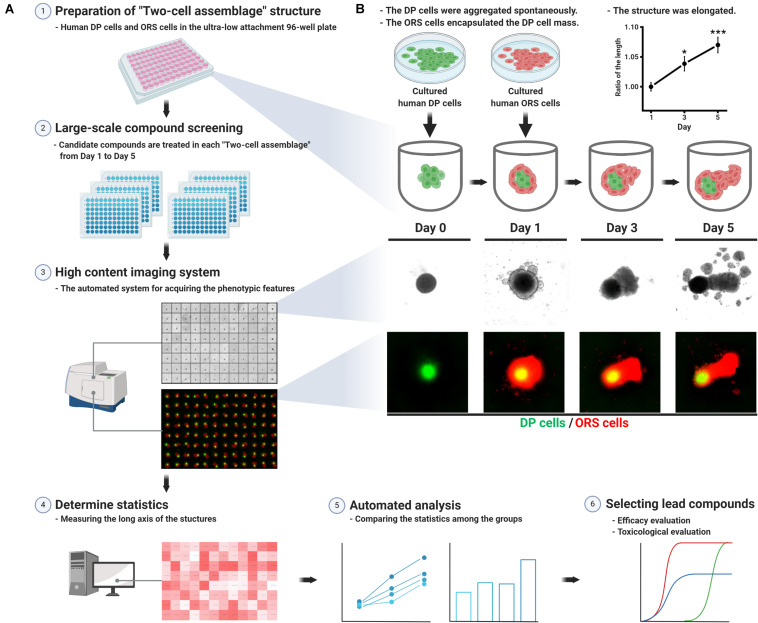
Schematic work flow for preparing the “two-cell assemblage (TCA)” structure and screening hair growth-promoting compounds. **(A)** Each TCA was loaded into each well of the ultra-low attachment 96-well plate, in which candidate compounds were treated for 5 days. The long axis length of the TCAs was acquired using a high content imaging system. The data for each candidate compound-treated group were compared to reveal the most effective compound, which would then require further evaluation. **(B)** Preparation of the TCA consisting of human dermal papilla (hDP) and human outer root sheath (hORS) cells, which was elongated for 5 days. The long axis length was measured without any treatment and compared with the culture day time points (right upper panel, data are presented as mean ± SEM and were evaluated using one-way analysis of variance with Tukey’s multiple comparisons test. *p < 0.05 or ***p < 0.001, compared to day 1).

The TCA assay employed herein is a simple *in vitro* method for screening hair growth-promoting compounds. Each TCA structure was prepared in an ultra-low attachment 96-well plate (Figure 1A), in which the two kinds of human cells (3D co-cultured hDP and hORS) constituted a unipolar elongated structure (Figure 1B). In detail, the cultured hDP cells (passage 2 or 3; 5 × 10^3^ cells/well) were combined in a single cell suspension and seeded in each well of the 96-well plate in Dulbecco’s Modified Eagle’s Medium supplemented with 10% fetal bovine serum and an antibiotic/antimycotic solution. The seeded hDP cells amassed and aggregated into a spherical structure in each well on day 0. On day 1, we added cultured hORS cells (passage 2 or 3; 5 × 10^3^ cells/well) into each well, replacing the media with William’s E medium (Gibco) supplemented with 2 mM L-glutamine, 10 ng/mL hydrocortisone, and 10 μg/mL insulin. From days 1 to 5, culture media supplemented with each hair growth-promoting molecule at the designated concentration was used for the TCA culture and changed every other day (Figure 1B). All procedures were undertaken at 37°C in a 5% CO_2_ atmosphere.

### Hematoxylin/Eosin (H&E) and Immunofluorescence Staining

Two-cell assemblage structures were mounted in Tissue-Tek cryo-OCT compound (Thermo Fisher) and frozen on dry ice. The frozen blocks were sectioned at a thickness of 8 μm at −25°C and were stained with hematoxylin/eosin (H&E). For immunofluorescence staining, the cryosections were incubated at 4°C overnight in a primary mouse antibody against human alkaline phosphatase (ALP) or Keratin 14 (K14), diluted in diluent reagent (Invitrogen). The nuclei were counterstained with 4′,6-diamidino-2-phenylindole (DAPI; Invitrogen). The stained slides were observed and photographed under a Nikon Eclipse Ci-L microscope or a Nikon Eclipse Ni-E fluorescence microscope (Nikon).

### Quantitative Real-Time Polymerase Chain Reaction (qPCR)

Experiments were performed by pooling 48 TCA structures for each quantitative real-time polymerase chain reaction (qPCR) experiment. Total RNA was isolated from the collected TCA structures 24 h after each hair growth-promoting molecule was used, using RNA iso Plus (TaKaRa Bio) and DNase I (Roche Pharmaceuticals). We used 1 μg of total RNA for the cDNA synthesis using a First Strand cDNA Synthesis Kit (Fermentas), according to the manufacturer’s instructions. The qPCR was performed in a 7500 Real-Time PCR System (Applied Biosystems) using SYBR Premix Ex Taq (TaKaRa Bio), according to the manufacturer’s instructions. The primer sequences are shown in Table 1. All experiments were performed in triplicate and were independently repeated three or four times. Data are presented as fold change relative to a control group, which was normalized to GAPDH expression.

**TABLE 1:** Primer sequence for real-time polymerase chain reaction.

**Human VEGFA**	
Sense	ACT TCT GGG CTG TTC TCG
Antisense	TCC TCT TCC TTC TCT TCT TC
**Human HGF**	
Sense	CGC AGC TAC AAG GGA ACA GT
Antisense	TCC TGT AGG TCT TTA CCC CGA
**Human IGF-1**	
Sense	TTC AAC AAG CCC ACA GGG
Antisense	GGT GCG CAA TAC ATC TCC
**Human FGF-7**	
Sense	TTG TGG CAA TCA AAG GGG TG
Antisense	CCT CCG TTG TGT GTC CAT TT
**Human FGF-10**	
Sense	TTC AAG GAG ATG TCC GCT
Antisense	GAT GCT GTA CGG GCA GTT
**Human PDGFA**	
Sense	GCC CAT TCG GAG GAA GAG
Antisense	TTG GCC ACC TTG ACG CTG CG
**Human PDGFB**	
Sense	GAA GGA GCC TGG GTT CCC
Antisense	TTT CTC ACC TGG ACA GGT

### Image Acquisition for TCA and Statistical Analysis

A high content screening system (ImageXpress Micro Confocal High-Content Imaging System, Molecular Devices) was used for TCA image acquisition from day 1 to 5, every other day (Figure 1). Quantification of the long axis length of the TCA structures was performed automatically, based on the statistical analysis being employed. All statistical analyses were performed in the Prism 8 software package (GraphPad Software), using the Kruskal–Wallis test with a *post hoc* test, one-way analysis of variance with a *post hoc* test, or two-way analysis of variance with a *post hoc* test. All tests were two-tailed, and *p* < 0.05 was considered statistically significant.

## Results

### TCA Structures Configured Spontaneously and Elongated in a Unipolar Manner Over 5 Days

The hDP cells seeded in the ultra-low attachment 96-well plate spontaneously aggregated into a 3D spherical structure within 24 h. The spheroidal hDP cellular mass was surrounded by hORS cells, which were subsequently seeded, resulting in a TCA structure (Figure 1B). The TCA became elongated in a unipolar manner over the 5 days of the study, with a statistically significant difference in the long axis length on day 5 compared to day 1 (Figure 1B, upper right panel). The hORS cells in the TCA constituted the elongated portion of the structure (Figure 1B).

### The Microstructure of the TCA *in vitro*

**FIGURE 2:**
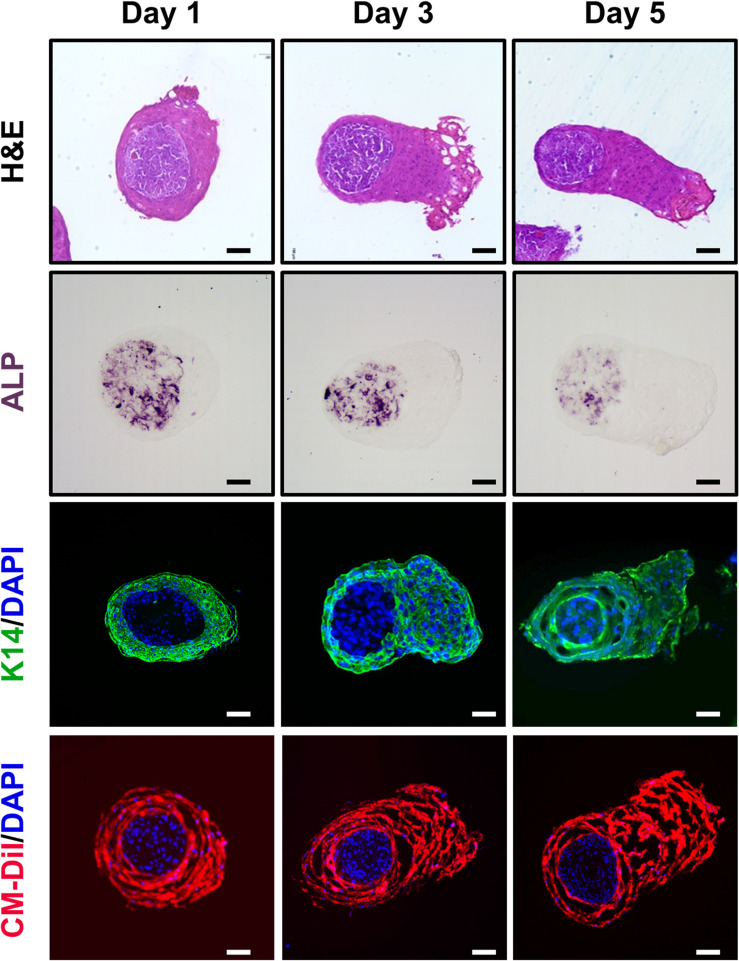
Histological characterizations of the “two-cell assemblage (TCA)” structure. Microscopic images of the TCA were visualized at days 1, 3, and 5 of culture, using hematoxylin and eosin stain, immunohistochemical stain (alkaline phosphatase, ALP), or immunofluorescence stain (keratin 14, K14; cell-labeling dye, CM-Dil). Bar: 100 μm.

Microscopic observation of the H&E stained TCA revealed spatial segregation in the inner cell mass and the outer shell cell layer (Figure 2). To check whether the inner cell mass was composed of hDP cells, we performed immunohistochemical staining for ALP, a signature marker of DP cells (Yang and Cotsarelis, 2010). Indeed, ALP was detected in the inner cellular mass, indicating that the amassed cells were hDP cells, as they mimicked the DP structure of an intact hHF organ (Figure 2). Immunofluorescent staining for K14 confirmed that the cells in the outer shell cell layer and the elongated portion of the TCA were hORS cells (Stark et al., 1987) (Figure 2). Furthermore, we used a cell tracking method to check the origin of the inner cell mass and outer shell cell layer. Green and red fluorescent colors were detected in the inner mass and the outer layer, respectively, confirming that our cultured hDP and hORS cells constituted the TCA structure (Figures 1B, 2).

### Hair Growth-Associated Gene Expression in the TCA Increased After Treatment With Hair Growth-Promoting Molecules

**FIGURE 3:**
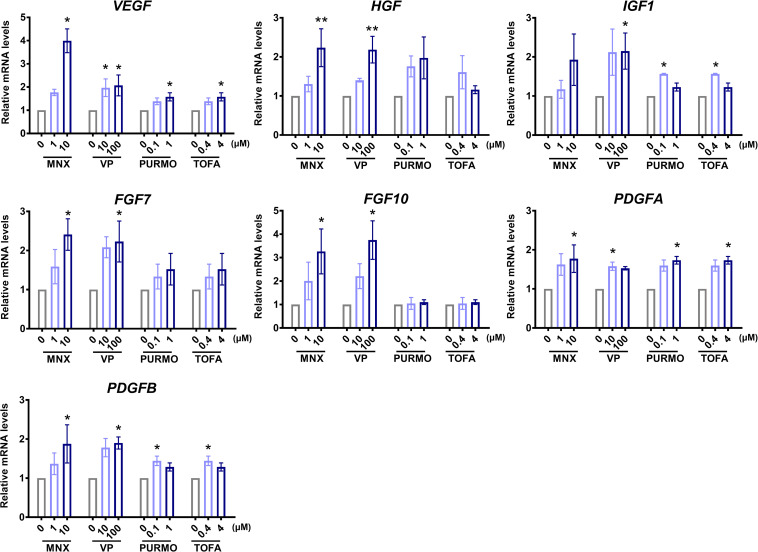
Characterizations of biological functionality in the “two-cell assemblage (TCA)” structure. Quantitative analysis of changes in the expression of hair growth-associated genes of the TCA after treatment with minoxidil (MNX), valproic acid (VP), purmophamine (PURMO), or tofacitinib (TOFA). The expression levels of genes were normalized to levels of the reference gene, GAPDH. Data are presented as mean ± SEM and were evaluated using the Kruskal–Wallis test with Dunn’s multiple comparison test.*p < 0.05 or **p < 0.01, compared to each control group (0 μM). Three or four independent experiments were carried out for each condition.

To evaluate the biological integrity of the TCA structure consisting of hDP and hORS cells, we measured the expression of hair growth-associated genes using real-time PCR, after treatment with well-established hair growth-promoting molecules, namely, MNX (Messenger and Rundegren, 2004), valproic acid (Jo et al., 2014), purmophamine (Paladini et al., 2005), and tofacitinib (Harel et al., 2015). If treatment of the established HF growth promotors on the TCA induced notable changes in the expression of genes associated with regulating HF growth or cycling, it could be inferred that the TCA would function as a suitable model for a HF mimetic. Among the hair growth-associated genes, we checked the mRNA expression levels of vascular endothelial growth factor (VEGF) (Yano et al., 2001), hepatocyte growth factor (HGF) (Shimaoka et al., 1995), insulin-like growth factor 1 (IGF1) (Philpott et al., 1994), fibroblast growth factor (FGF) 7 (Iino et al., 2007), FGF10 (Jang, 2005), and platelet-derived growth factor (alpha and beta; PDGFA and PDGFB) (Tomita et al., 2006) in the TCA (Figure 3). Indeed, most of the investigated genes showed increased expression levels with statistical significance after treatment with the hair growth-promoting molecules (Figure 3). Thus, we confirmed that the TCA maintained biological functional integrity when reacting to hair growth-promoting molecules.

### The Phenotypic Feature of the TCA as an Indicator of Hair Growth-Promoting Potential

**FIGURE 4:**
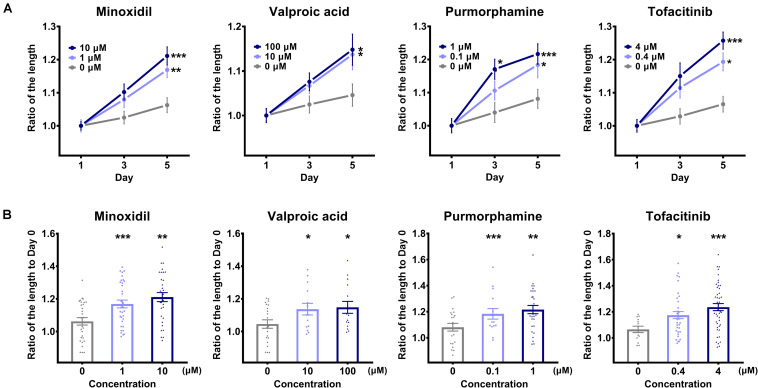
Comparison of the long axis length of the “two-cell assemblage (TCA)” structure. **(A)** The long axis length of the TCA was compared after treatment with each molecule, with designated concentrations on the TCA structures, across three time points. Data are presented as mean ± SEM and were evaluated using one-way analysis of variance with Tukey’s multiple comparisons test. *p < 0.05, **p < 0.01, or ***p < 0.001, compared to each control group on day 1. **(B)** The long axis length of the TCA was compared on day 5 among the different concentrations of each molecule. Data are presented as mean ± SEM and were evaluated using one-way analysis of variance with Tukey’s multiple comparisons test. *p < 0.05, **p < 0.01, or ***p < 0.001, compared to each control group (0 μM).

Based on the findings that the TCA was elongated in the *in vitro* culture environment and had intact biological functionality, we observed its phenotypic features every other day during treatment with the hair growth-promoting molecules, paying particular attention to the long axis length. We used a micro confocal high-content imaging system, which could provide reproducible and reliable results in a timely manner when carrying out statistical analyses. Interestingly, we observed that the degree of TCA elongation increased with increasing treatment of the hair growth-promoting molecules (Figure 4A). On the fifth day of culture, for all molecules, we observed a statistically significant difference in the length of the TCA compared to each control group, revealing that the TCAs in groups treated with hair growth-promoting molecules were elongated prominently in a dose-dependent manner, compared to those in the control group (Figure 4A). The long axis length of the TCA on day 5 (Figure 4B) serves as an indication of the hair growth-promoting potential of the candidate molecule.

## Discussion

To date, several methods for evaluating the hair growth-promoting effects of candidate compounds have been used, mainly based on hHF cells or the hHF organ (Yoo et al., 2010). Additionally, a human epidermal keratinocytes-based system measuring TGF-β1 promoter activity has been proposed (Huh et al., 2009). Although the hHF organ culture method has the advantage of being reliable (Philpott et al., 1996), its limited availability makes it difficult to screen many compounds. In order to evaluate and compare the hair growth-promoting efficacy of candidate substances in a large molecular library at once, an easily obtainable and accessible platform is necessary.

We established, herein, an hDP cellular mass structure as a backbone of the TCA assay. hDP cells are commercially available and can be easily cultured and mass produced. Indeed, *in vitro* assays using hDP cells have been widely used as validating models for hair growth-promoting molecules (Madaan et al., 2018), based on the fact that DP play crucial roles in hair growth and HF regeneration by regulating HF cells (Yang and Cotsarelis, 2010; Lei et al., 2017b). In this assay, hDP cells were observed to have spontaneously aggregated into a spherical structure within 24 h, which is consistent with previous studies (Higgins et al., 2010; Huang et al., 2013; Topouzi et al., 2017). The cellular properties of 3D spheroid cultured hDP cells were different from those observed for cultured cells in a 2D culture environment (Higgins et al., 2010). These differences include enhanced expression profiles of key DP signature genes (Gupta et al., 2018; Tan et al., 2019), and HF inductive ability (Kang et al., 2012; Higgins et al., 2013), suggesting that 3D cultured hDP cells are more similar, having exhibited physiologic intact DP status *in vivo*. Consequently, 3D cultured hDP spheroid-based drug assay systems that employ direct quantitation of DP cell proliferation or enhanced gene expression of growth factors and extracellular matrix proteins as efficacy indicators after treatment with hair growth-modulating molecules have been suggested (Lim et al., 2013; Gupta et al., 2018).

However, hDP cells alone cannot fully reflect the HF growth-promoting effect of candidate molecules, as HF is a mini-organ consisting of epithelial and mesenchymal components that interact reciprocally (Rendl et al., 2005; Chan et al., 2015; Huang et al., 2017). Accordingly, we grafted hORS cells into the 3D spheroid hDP cell mass structures to mimic the epithelial part of the HF, maintaining the spatial separation of two kinds of cells, thus resembling HF *in vivo*. We observed that the dissociated hORS cells encapsulated the hDP cell mass without any manipulation. Indeed, it has previously been found that dissociated skin cells have the potential to undergo a self-organization process (Lei et al., 2017a; Weber et al., 2019). The inner core–outer shell micro structures with unipolar orientation were not limited to our hORS and DP cell co-culture model. A recent study found that a keratinocyte and hDP cell co-culture system formed a similar structure with migratory polarization conserving central hDP cell aggregation (Tan et al., 2019). In another study attempting to establish an *in vitro* 3D organoid model, DP spheroids with a silk-gelatin were encapsulated by HF epithelial cells in a co-culture system (Gupta et al., 2018). Furthermore, hDP cell aggregates cultured with epithelial cells more prominently expressed DP-specific signature genes than those without epithelial cells (Gupta et al., 2018; Kageyama et al., 2019). Collectively, using a 3D aggregation of hDP cells co-cultured with hORS cells would be a reasonable methodology mimicking a physiologic HF organ *in vivo*, as inaugurated in our TCA assay for screening the hair growth-promoting effects of candidate compounds.

Previously, Havlickova et al. (2009) proposed a simplified HF-like 3D *in vitro* system for discovering hair growth-promoting drugs, consisting of hDP and hORS cells within an extracellular matrix. Their system used immunofluorescent staining-based findings for Ki67+ or TUNEL+ cells in the folliculoid microsphere structure as endpoints for determining the HF growth potential of candidate compounds. While this is a reasonably well implemented system, an additional step for staining each microsphere structure is needed to quantify the immunofluorescent signals. Herein, we obtained screening results based only on the length of the polar elongated TCA structures, without any further evaluation. Using length as an indicator for screening the hair growth-promoting effects can be optimized by complementing it with a high-throughput automated confocal microscopy platform, which is widely used for rapid large scale drug discovery (Dorval et al., 2018). In this way, we further cut down the labor and time demands required to measure the statistics, relative to the manual quantifying process (Figure 1A). Our phenotypic screening system would provide researchers with a high-throughput system with automated analytic processing of raw datasets, enabling the identification of the most promising molecule among a group of candidate molecules.

In this TCA assay, we used two types of hHF-constituting cells: hDP and hORS. However, the hair growth cycle is affected by the *in vivo* microenvironment around the hHFs, considering that each *in vivo* hHF organ interacts with neighboring cells, such as fibroblasts, endothelia, immunocytes, melanocytes, nerve cells, and adipocyte, as well as the extracellular matrix (Chen et al., 2016; Fan et al., 2018; Chen et al., 2020). Hence, using two kinds of hHF constituent cells, as done in this method, would not be enough to fully reflect the *in vivo* microenvironment (Abaci et al., 2017). Indeed, a combination of an outer adipose-derived stem cell shell and an inner DP core has been shown to exhibit superior DP signatures compared to DP cells alone (Huang et al., 2016). It is therefore necessary to develop an *in vitro* assay system integrating other types of cells and extracellular structures to simulate the *in vivo* hHF in the future.

Nevertheless, the advantages of this assay are: (i) it is physiologically and practically advantageous as it uses 3D cultured two-type human cells, which are easily available; (ii) it is simple as it uses length as the only endpoint; (iii) the presence of a high throughput system, which screens various compounds simultaneously. In conclusion, the “TCA” assay could serve as a method to validate the hair growth-promoting effect of a large volume of library molecules, although this assay needs to be validated further.

## Data Availability Statement

The raw data supporting the conclusions of this article will be made available by the authors, without undue reservation.

## Ethics Statement

The studies involving human participants were reviewed and approved by the Institutional Review Board of Seoul National University. The patients/participants provided their written informed consent to participate in this study.

## Author Contributions

SJ, JO, and OK conceived and planned the experiments. SJ, BMK, and MP carried out the experiments. SJ, JO, KHK, and OK contributed to the interpretation of the results. SJ and JO took the lead in writing the manuscript. All authors provided critical feedback and helped shape the research, analysis, and manuscript. OK supervised the project.

## Conflict of Interest

The authors declare that the research was conducted in the absence of any commercial or financial relationships that could be construed as a potential conflict of interest.
